# The effect of *ABCA1 *gene polymorphisms on ischaemic stroke risk and relationship with lipid profile

**DOI:** 10.1186/1471-2350-8-30

**Published:** 2007-06-06

**Authors:** Alireza Pasdar, Ghasem Yadegarfar, Alastair Cumming, Lawrence Whalley, David St Clair, Mary-Joan MacLeod

**Affiliations:** 1 Department of Mental Health, University of Aberdeen, UK; 2 Public Health Sciences & Medical Statistics Group, University of Southhampton, UK; 3 Department of Molecular and Cell Biology, University of Aberdeen, UK; 4 Department of Medicine and Therapeutics, University of Aberdeen, UK

## Abstract

**Background:**

Ischaemic stroke is a common disorder with genetic and environmental components contributing to overall risk. Atherothromboembolic abnormalities, which play a crucial role in the pathogenesis of ischaemic stroke, are often the end result of dysregulation of lipid metabolism. The ATP Binding Cassette Transporter (*ABCA1*) is a key gene involved in lipid metabolism. It encodes the cholesterol regulatory efflux protein which mediates the transfer of cellular phospholipids and cholesterol to acceptor apolipoproteins such as apolipoprotein A-I (ApoA-I). Common polymorphisms in this gene affect High Density Lipoprotein Cholesterol (HDL-C) and Apolipoprotein A-I levels and so influence the risk of atherosclerosis. This study has assessed the distribution of *ABCA1 *polymorphisms and haplotype arrangements in patients with ischaemic stroke and compared them to an appropriate control group. It also examined the relationship of these polymorphisms with serum lipid profiles in cases and controls.

**Methods:**

We studied four common polymorphisms in *ABCA1 *gene: G/A-L158L, G/A-R219K, G/A-G316G and G/A-R1587K in 400 Caucasian ischaemic stroke patients and 487 controls. Dynamic Allele Specific Hybridisation (DASH) was used as the genotyping assay.

**Results:**

Genotype and allele frequencies of all polymorphisms were similar in cases and controls, except for a modest difference in the *ABCA1 *R219K allele frequency (P-value = 0.05). Using the PHASE2 program, haplotype frequencies for the four loci (158, 219, 316, and 1587) were estimated in cases and controls. There was no significant difference in overall haplotypes arrangement in patients group compared to controls (p = 0.27). 2211 and 1211 haplotypes (1 = common allele, 2 = rare allele) were more frequent in cases (p = 0.05). Adjusted ORs indicated 40% and 46% excess risk of stroke for these haplotypes respectively. However, none of the adjusted ORs were statistically significant. Individuals who had R219K "22" genotype had a higher LDL level (p = 0.001).

**Conclusion:**

Our study does not support a major role for the *ABCA1 *gene as a risk factor for ischaemic stroke. Some haplotypes may confer a minor amount of increased risk or protection. Polymorphisms in this gene may influence serum lipid profile.

## Background

Ischaemic stroke is a complex disorder. Hypertension and dyslipidemia are known to influence atherosclerosis and so predispose to ischaemic stroke. Hypercholesterolemia increases the risk of stroke [1], while lipid lowering therapy significantly reduces risk of stroke [2]. Low HDL has also been reported as a risk factor for ischaemic stroke [3,4]. Several genes involved in lipid metabolism such as Paraoxonase (*PON*), Lipoprotein Lipase (*LPL*) and Fatty Acid-Binding Protein 2 (*FABP2*) have been examined in the stroke populations [5-8]. ApoE protein is involved in cholesterol transport and some studies suggest ApoE *ε 4 and ApoE *ε 2 alleles are associated with higher risk and lower risk of ischaemic stroke respectively, but this has not been confirmed in all studies [9,10].

Homozygous mutations including deletions and polymorphisms of the ATP-binding cassette transporter 1 *(ABCA1) *cause Tangier disease, a rare autosomal recessive disorder with congenital HDL deficiency and increased risk of atherosclerosis. Heterozygous mutants develop familial HDL deficiency in which less severe HDL deficiency is associated with reduced cholesterol efflux, which also predispose to atherosclerosis [11-13]. In the general population, low plasma HDL cholesterol (HDL-C) increases the risk of coronary artery disease (CAD) [14-16]. Variations in *ABCA1 *gene and alteration in gene expression have been shown to affect the risk of atherosclerosis in animal models [17,18] and invitro studies show that *ABCA1 *also modulates LDL oxidation in the artery wall cells [19]. *ABCA1 *gene expression is upregulated in atherosclerotic plaques, further suggesting an association between *ABCA1 *and atheroma [20]. Reports have suggested a relationship between individual polymorphisms of *ABCA1 *gene and lipid profiles [21,22]. Furthermore, ABCA1 appears to be required in neuronal tissue for cholesterol efflux to ApoA-I and ApoE. ABCA1 deficiency in the brain causes a dramatic fall in neuronal ApoE levels [23,24]. The only published study in ischaemic stroke on 244 Hungarian patients [25] suggests a protective role for the *ABCA1*-R219K and V771M polymorphisms. The aim of this study was to assess the distribution of different polymorphisms and haplotype arrangements of the *ABCA1 *gene, and any association with lipid profile, in a series of well-phenotyped ischaemic stroke patients and unaffected control subjects.

## Methods

### Subjects

After ethical committee approval and informed consent, 400 Caucasian patients admitted to a regional hospital with a CT confirmed diagnosis of ischemic stroke were recruited over a period of 3 years. Four hundred and eighty seven Caucasian individuals from the same region, without any clinical cerebrovascular disease were used as controls. All were volunteers, 194 were part of a birth cohort (Aberdeen Birth Cohort) born in 1921. The others were recruited through general practices, so that overall controls were roughly age matched to cases. For a subgroup of controls (n = 181) detailed lipid parameters including total cholesterol, VLDL-Cholesterol, VLDL-Triglyceride, IDL, LDL, HDL2, HDL3 were available for comparison with *ABCA1 *genotypes.

### Classification of stroke patients

On the basis of clinical, laboratory and radiological parameters, all cases were assessed by an experienced stroke physician according to Trial of Org 10172 in Acute Stroke Treatment (TOAST) criteria [26] to determine stroke subtype (Table 2). Haemorrhagic stroke cases were not included. For most patients lipid levels were checked either at admission or the following morning. No patients included in this analysis were known to be on statin on admission.

### Genotyping method and markers

DNA was extracted using Nucleon^® ^DNA extraction kits. 5–10ng DNA was amplified through Hot Start PCR protocol using Immolase Taq DNA polymerase^®^. We used different sets of primers for each SNP as follows:

**1- *ABCA1*-rs2230805 **(G/A; L158L); designated as **[*ABCA1*-6]**

*ABCA1*-2230805F: 5'-biotin-TAGACTTTGGGAGAGAGAGGTTGT-3'

*ABCA1*-2230805R: 5'-AATGAAACCTTCTCTGGGTTCC-3'

**2- *ABCA1*-rs2234884 **(G1051A; R219K); designated as **[*ABCA1*-3]**

*ABCA1*-2234884F: 5'-biotin-AATTTCTGAGCTTTGTGGACTA-3'

*ABCA1*-2234884R: 5'-GCTCTGCTGCAGTCATTTTCTC-3'

**3- *ABCA1*-rs2246841 **(G888A; G316G); designated as **[*ABCA1*-5]**

*ABCA1*-2246841F: 5'-biotin-TACCAGTTGAGAGACTTGATCTTC-3'

*ABCA1*-2246841R: 5'-TCTCGTATTGTCTGTGGGCATC-3'

**4- *ABCA1*-rs1997618 **(c/t, T1555I)

*ABCA1*-**1997618**F: 5'-biotin-CCGAGTCAAGAAGTTAATGATGC-3'

*ABCA1*-**1997618**R: 5'-GCTTTAGGTGTTTCTTCATTTGTTT-3'

**5- *ABCA1*-rs2234886 **(G5155A; R1587K); designated as **[*ABCA1*-4]**

*ABCA1*-2234886F: 5'-biotin-CAGCGGTTTACCTTGACATTATT-3'

*ABCA1*-2234886R: 5'-GAAGATTTATGACAGGACTGGACAC-3'

**6- *ABCA1*-rs1883024 **(c/t, P1648L)

*ABCA1*-**1883024**F: 5'-biotin-TATACAGACACAGCCACACTTA-3'

*ABCA1*-**1883024**R: 5'-ATCTCACCAAGCAGCAGTTCTC-3'

Of the six *ABCA1 *SNPs, only four SNPs (G/A-L158L, G/A-R219K, G/A-G316G and G/A-R1587K) were polymorphic in the Scottish population. All had genotype frequencies in Hardy Weinberg equilibrium. Genotyping assays for determining different alleles were based on the Dynamic Allele Specific Hybridisation (DASH) technique as described by the developers [27]. Genotype scoring in this method is based on the melting temperature curves of the amplified DNA strand containing the polymorphic site and its complementary specific probe in presence of a dye.

### Data analysis

Association between markers and disease phenotype was tested with Pearson χ^2 ^test. To explore distribution of categorical data, hypertension and gender, between cases and controls, chi-square test was used. Lipid parameters were log transformed before analysis. Numerical covariates, age, total cholesterol, HDL, LDL, total triglyceride and glucose, were compared between cases and controls by t-test. Smoking status was incomplete for a subgroup of controls and therefore not included in analysis. A univariate logistic regression method was carried out in order to identify factors potentially associated with risk of stroke. To control for the effect of confounding factors and covariates, multiple logistic regression with SPSS version 14 (Statistical Package for Social Science) was used for statistical analysis. A p-value of less than or equal to 0.05 was considered statistically significant.

ANOVA was used for comparing the effect of different genotypes on lipid profiles. Independent samples T-test was also used to compare means of different groups based on quartiles or carrier status. Haplotype frequencies for the four loci (G/A-L158L, G/A-R219K, G/A-G316G and G/A-R1587K) were estimated for cases and controls, using PHASE Ver.2.11 [28].

## Results

Demographic parameters and serology results are shown in Table 1.

**Table 1:** Specifications of cases and controls

	Cases (n = 400)	Controls (n = 487)
Mean age (± SD)^§^	66 (± 11)	64.2(± 12.3)
Sex (M:F Ratio)^§^	1.4	0.97
Hypertension*^§^	41%	18%
Diabetes Mellitus**^§^	12.2%	3.2%
Total Cholesterol mmol/l (Ave., SD)^§^	5.6 (± 1.3)	5.8 (± 1.2)
HDL mmol/l (Ave., SD)^§^	1.2 (± 0.4)	1.4 (± 0.4)
LDL mmol/l (Ave., SD)	3.5 (± 1.2)	3.5 (± 1.1)
Total Triglyceride mmol/l (Ave., SD)	1.7 (± 1.1)	1.8 (± 1.1)
Glucose mmol/l^§^	6.4 (± 2.4)	5.9 (± 1.2)

* Hypertension defined as blood pressure > 160/90 mmHg or history of hypertension/on hypertensive therapy

** History of diabetes or confirmed laboratory diagnosis

§Significant difference (P < 0.05) between cases and controls

**Table 2:** Different stroke subtypes based on TOAST classification

**Subtype**	**All Stroke Samples**	
	
	Number	%
Large Vessel Disease (LVD)	141	35.3%
Small Vessel Disease (SVD)	98	24.5%
Cardioembolic	76	19%
Other/Unclear	85	21.3%
Hemorrhagic Stroke	0	0

TOTAL	400	100%

### Allelic distributions and genotype frequencies

*ABCA1*-6 (G/A; L158L) "G" allele frequency (L variant) in cases and controls was 69.7% and 72.3% respectively (p = 0.25; OR = 0.88, 95%CI: 0.71–1.09). *ABCA1*-3 (G1051A; R219K) "G" allele frequency (R variant) was 68.2% in cases and 73.2% in controls (p = 0.05; OR = 0.79, 95%CI: 0.62–1.00), *ABCA1*-5 (G/A; G316G) "G" allele frequency (G variant) was 88% in cases and 86.7% in controls (p = 0.42; OR = 1.13, 95%CI: 0.84–1.52); *ABCA1*-4 (G5155A; R1587K) "G" allele frequency (R variant) was 77.4% in cases and 75.9% in controls (p = 0.47; OR = 1.09, 95%CI: 0.86–1.37). After adjusting for confounding factors and covariates, minor changes in the risk were not statistically significant.

In addition to a trend to lower *ABCA1*-3 (G1051A; R219K) "G" allele frequency in all cases (p = 0.05), especially in males (p = 0.04), the "22" genotype was higher in LVD cases (p = 0.04) and also in patients over 65 years (p = 0.01), compared to controls. *ABCA1*-5 (G/A; G316G) "11" genotype was also commoner in cases than in controls (p = 0.04). However after adjusting OR for confounders the difference did not remain significant.

There were no significant differences in allele frequencies between cases and controls in each gender group, or between stroke TOAST subgroups for the other SNPs tested.

### Effects of genotypes on lipid profile

We also studied the effects of different *ABCA1 *genotypes on lipid profile in all cases and controls. Cases with *ABCA1*-3 (G1051A; R219K) "22" genotype had a higher LDL level compared to controls which remained significant after Bonferroni correction (p = 0.003). In 181 of the controls who had a more detailed lipid profile, no differences were found in VLDL-Cholesterol, IDL, LDL, HDL2, HDL3, total cholesterol and triglyceride levels in females or males between different genotypes of *ABCA1*. However, in this subgroup, females with *ABCA1*-3 (G1051A; R219K) "11" and *ABCA1*-4 (G5155A; R1587K) "22" genotypes had a higher level of IDL (p = 0.01 and p = 0.02) respectively.

### Effects of alleles on lipid profile

Comparing the lipid profiles in carriers of different *ABCA1 *alleles revealed that individuals who were the *ABCA1*-R219K carrier (K allele) had a lower TG level than non carriers (p = 0.005). This trend was observed in cases (p = 0.017) but not in controls (p = 0.8). The lipid profile of the population in the presence of different *ABCA1 *gene alleles has been summarised in tables 3.

**Table 3:** lipid profiles changes in different ABCA1 genotypes and allele carriers:

A. Lipid profile in carriers of different ABCA1 gene alleles in the *whole population *(cases and controls altogether)							
		Carrier 1 (11+12)	Non car. (22)	p-value	Carrier 2 (22+12)	Non car. (11)	p-value

ABCA1-6	HDL	1.3597	1.3605	NS	1.3655	1.3545	NS
	LDL	3.4775	3.5805	NS	3.4439	3.5273	NS
	TC	5.6951	5.8002	NS	5.6163	5.7877	NS
	TG	1.7831	1.7436	NS	1.6834	1.8690	**0.017**
							
ABCA1-3	HDL	1.3446	1.2960	NS	1.3566	1.3254	NS
	LDL	3.5526	3.7406	NS	3.5246	3.6108	NS
	TC	5.7511	5.7903	NS	5.6764	5.8295	NS
	TG	1.7837	1.6215	NS	1.6546	1.8804	**0.005**
							
ABCA1-5	HDL	1.3587	1.4355	NS	1.4073	1.3456	NS
	LDL	3.4945	3.4193	NS	3.4848	3.4956	NS
	TC	5.7044	5.6052	NS	5.6795	5.7095	NS
	TG	1.7832	1.5616	NS	1.6355	1.8233	**0.014**
							
ABCA1-4	HDL	1.3658	1.2838	NS	1.3338	1.3818	NS
	LDL	3.4783	3.6276	NS	3.5464	3.4413	NS
	TC	5.6993	5.7631	NS	5.7841	5.6434	NS
	TG	1.7690	1.7868	NS	1.7727	1.7679	NS

**B. **Lipid profile in carriers of different ABCA1 gene alleles in *controls*							

ABCA1-6	HDL	1.4429	1.4804	NS	1.4578	1.4350	NS
	LDL	3.4442	3.4792	NS	3.4147	3.4762	NS
	TC	5.8044	5.9003	NS	5.7374	5.8794	NS
	TG	1.8046	1.7877	NS	1.7099	1.8881	NS
							
ABCA1-3	HDL	1.4416	1.4097	NS	1.4681	1.4143	NS
	LDL	3.4805	3.5634	NS	3.4173	3.5464	NS
	TC	5.8415	5.8622	NS	5.7426	5.9319	NS
	TG	1.8264	1.7660	NS	1.7310	1.9042	NS
							
ABCA1-5	HDL	1.4473	1.3916	NS	1.4920	1.4311	NS
	LDL	3.4534	3.5611	NS	3.4592	3.4540	NS
	TC	5.8155	5.6472	NS	5.7071	5.8175	NS
	TG	1.8143	1.4965	NS	1.7026	1.8433	NS
							
ABCA1-4	HDL	1.4528	1.3398	NS	1.4143	1.4712	NS
	LDL	3.4343	3.5858	NS	3.5336	3.3742	NS
	TC	5.7987	5.8264	NS	5.8773	5.7429	NS
	TG	1.7965	1.8134	NS	1.7893	1.8036	NS

**C. **Lipid profile in different carriers of ABCA1 gene alleles in *cases*							

ABCA1-6	HDL	1.2357	1.2333	NS	1.2362	1.2348	NS
	LDL	3.5242	3.6879	NS	3.4813	3.6015	NS
	TC	5.4888	5.7000	NS	5.4662	5.6623	NS
	TG	1.7499	1.6923	NS	1.6447	1.8395	NS
							
ABCA1-3	HDL	1.2123	1.2107	NS	1.2217	1.2024	NS
	LDL	3.6464	3.8690	NS	3.6485	3.7008	NS
	TC	5.6403	5.7424	NS	5.6057	5.7004	NS
	TG	1.7236	1.5037	NS	1.5596	1.8459	**0.017**
							
ABCA1-5	HDL	1.2319	1.4857	NS	1.2691	1.2288	NS
	LDL	3.5502	3.2571	NS	3.5243	3.5494	NS
	TC	5.5619	5.5571	NS	5.5079	5.5766	NS
	TG	1.7372	1.6483	NS	1.5243	1.7948	**0.016**
							
ABCA1-4	HDL	1.2452	1.1867	NS	1.2169	1.2603	NS
	LDL	3.5358	3.7000	NS	3.5640	3.5282	NS
	TC	5.5760	5.6533	NS	5.6622	5.5212	NS
	TG	1.7292	1.7407	NS	1.7480	1.7166	NS

### Haplotypes

Haplotype analysis was performed using PHASE version 2.11. The PHASE program [28], resulted in reconstruction of 16 different haplotypes in cases and controls. Overall haplotype frequencies in cases and controls were not reported significantly different by the program (p = 0.27), although there were differences in specific haplotypes between the groups. Adjusted ORs indicated 40%, 27% and 46% excess risk of stroke for haplotypes 2211, 2212 and 1211 respectively. However, none of these risks were statistically significant (Table 4). The haplotypes arrangements did not influence the lipid profile of cases or controls.

**Table 4:** Haplotypes frequencies in cases and controls

Haplotype	Controls No. (%)	Cases No. (%)	Crude OR (95% CI)	p-value for crude OR	Adjusted* OR (95% CI)	p-value for adjusted OR
1111	591 (60.68)	463 (57.88)	0.89 (0.74–1.08)	0.232	0.94 (0.75–1.19)	0.611
1112	92 (9.45)	75 (9.38)	0.99 (0.72 – 1.37)	0.960	0.94 (0.64 – 1.38)	0.751
2211	80 (8.21)	88 (11.00)	1.38 (1.01 – 1.90)	**0.047**	1.40 (0.95 – 2.07)	0.086
2222	56 (5.75)	31 (3.88)	0.66 (0.42 – 1.04)	0.071	0.75 (0.43 – 1.28)	0.284
2212	54 (5.54)	56 (7.00)	1.28 (0.87 – 1.89)	0.207	1.27 (0.79 – 2.04)	0.315
2221	52 (5.34)	55 (6.88)	1.31 (0.89 – 1.94)	0.177	1.12 (0.70 – 1.78)	0.650
1211	12 (1.23)	20 (2.50)	2.06 (1.00–4.23)	**0.050**	1.46 (0.61 – 3.53)	0.398
2111	10 (1.03)	7 (0.88)	0.85 (0.32 – 2.25)	0.744	0.63 (0.16 – 2.38)	0.492
Others^$^	27 (2.78)	5 (0.63)	0.22 (0.09 – 0.57)	**0.002**	0.29 (0.10 – 0.82)	0.020

Total Alleles	974	800				

* ORs adjusted for age, gender, hypertension, triglyceride, HDL, total cholesterol and diabetes.

$ Less frequent haplotypes

## Discussion

The *ABCA1 *gene is known to have a crucial role in lipid metabolism [29,30]. Mutations or polymorphisms in this gene are known to cause dyslipidemia such as low HDL-C and thus predispose to atherosclerosis [31,32]. Several polymorphisms of the *ABCA1 *gene have been investigated for their association with CAD [33-35]. Other works have reported that CAD patients who are carriers of R219K allele had less severe atherosclerosis [31] and overall lower risk of CAD [36]. Andrikovics *et al *recently reported a higher frequency of R219K in controls than in Hungarian stroke patients and suggested a protective role for this polymorphism [25]. By contrast, our control group had a higher "R219" allele frequency, while the stroke population had a higher "219K" allele frequency. The "219K" allele frequency is similar to that reported in other studies of Irish and other Scottish populations [36]. Two SNPs, P1648L and T1555I, were not polymorphic in our population.

While the R219K -G1051A- (A or "K" variant) has been associated with decreased TG, increased HDL and subsequently a lower risk for atherosclerotic progression, in contrast the R allele has been associated with vascular disease [31]. This has not been confirmed in our study, although in our stroke population those with R219K "22" genotype (AA) had a higher level of LDL and the "K allele" carriers had a lower TG. Clee *et al *also reported lower TG in the carriers of 219K variant and this finding was replicated in our population (p = 0.006). The HDL level showed no significant difference among different R219K genotypes.

It is also of interest that a protective role for the 219K allele has not been confirmed by all studies. Ethnic background or other environmental factors may weaken the link with HDL-C levels. However, in three other European populations in contrast to a Japanese one, R219 has been constantly the wild type allele [37].

Haplotype analysis can provide additional power in association studies in complex diseases [38]. Results using different programs are usually consistent, but sometimes there are minor variations [6,39]. We performed haplotype analysis in the remaining four SNPs, and only the 2211 and 1211 haplotypes were more frequent in cases (p = 0.05). Only a small proportion of individuals carried these haplotypes, thus the result should be interpreted with caution.

We found an association between LDL levels and *ABCA1 *genotype, but not with HDL. Epidemiological studies of *ABCA1 *polymorphisms and HDL levels suggest that only 10% of HDL level variation maybe explained by this gene [40] and thus our study may not have been large enough to detect this. Other studies have shown an association between the R219K polymorphism and MI, but no association between haplotype arrangements and MI. Polymorphisms in the promoter region (C-564T) and in the coding region (R1587K) have shown an association with ApoA-I levels but these have not been associated with vascular disease. Another study has suggested that rare alleles with major phenotypic effects contribute significantly to low plasma HDL [41]. Although lipid measurements were made early after admission, possible confounders include the acute lipid changes that occur after acute stroke. The lipid levels reported in our study are similar to those values on the morning after admission reported by Dyker, Weir and Lees in patients after acute stroke [42]. The changes in lipids post stroke remain controversial, but further studies of changes in lipid profile will be difficult because of the early introduction of statin therapy on the basis of studies such as SPARCL [43].

## Conclusion

In conclusion *ABCA1 *was not associated with ischaemic stroke in our population. Among the studied *ABCA1 *gene polymorphisms, R219K has the greatest impact on lipid profile especially LDL and TG. Large scale epidemiological studies may be necessary to definitively confirm an association of this gene with specific vascular diseases.

## Competing interests

The author(s) declare that they have no competing interests.

## Authors' contributions

AP participated in study design, carried out the genotyping and the statistical analyses and drafted the manuscript. GY reviewed and reanalysed the data and commented on statistics. AC helped with study design and commented on the manuscript. LW and DS contributed to the study design, sample collection and helped draft the manuscript. MJM contributed to study design, coordinated recruitment and drafted the manuscript. Authors read and approved the final manuscript.

## Pre-publication history

The pre-publication history for this paper can be accessed here:


